# Molecular cytogenetic characterisation of a mosaic add(12)(p13.3) with an inv dup(3)(q26.31 → qter) detected in an autistic boy

**DOI:** 10.1186/1755-8166-2-16

**Published:** 2009-08-04

**Authors:** Isabel M Carreira, Joana B Melo, Carlos Rodrigues, Liesbeth Backx, Joris Vermeesch, Anja Weise, Nadezda Kosyakova, Guiomar Oliveira, Eunice Matoso

**Affiliations:** 1 Laboratório de Citogenética, Instituto de Biologia Médica e Centro de Neurociências e Biologia Celular, Faculdade de Medicina, Universidade de Coimbra, Portugal; 2 Center for Human Genetics, Katholieke Universiteit Leuven, University Hospital Leuven, Belgium; 3 Jena University Hospital, Institute of Human Genetics and Anthropology, Kollegiengasse 10, D-07743 Jena, Germany; 4 Unidade Neurodesenvolvimento e Autismo, Hospital Pediátrico, Centro Hospitalar de Coimbra, Portugal

## Abstract

**Background:**

Inverted duplications (inv dup) of a terminal chromosome region are a particular subset of rearrangements that often results in partial tetrasomy or partial trisomy when accompanied by a deleted chromosome. Associated mosaicism could be the consequence of a post-zygotic event or could result from the correction of a trisomic conception. Tetrasomies of distal segments of the chromosome 3q are rare genetic events and their phenotypic manifestations are diverse. To our knowledge, there are only 12 cases reported with partial 3q tetrasomy. Generally, individuals with this genomic imbalance present mild to severe developmental delay, facial dysmorphisms and skin pigmentary disorders.

**Results:**

We present the results of the molecular cytogenetic characterization of an unbalanced mosaic karyotype consisting of mos 46,XY,add(12)(p13.3) [56]/46,XY [44] in a previously described 11 years old autistic boy, re-evaluated at adult age. The employment of fluorescence *in situ *hybridization (FISH) and multicolor banding (MCB) techniques identified the extra material on 12p to be derived from chromosome 3, defining the additional material on 12p as an inv dup(3)(qter → q26.3::q26.3 → qter). Subsequently, array-based comparative genomic hybridization (aCGH) confirmed the breakpoint at 3q26.31, defining the extra material with a length of 24.92 Mb to be between 174.37 and 199.29 Mb.

**Conclusion:**

This is the thirteenth reported case of inversion-duplication 3q, being the first one described as an inv dup translocated onto a non-homologous chromosome. The mosaic terminal inv dup(3q) observed could be the result of two proposed alternative mechanisms. The most striking feature of this case is the autistic behavior of the proband, a characteristic not shared by any other patient with tetrasomy for 3q26.31 → 3qter. The present work further illustrates the advantages of the use of an integrative cytogenetic strategy, composed both by conventional and molecular techniques, on providing powerful information for an accurate diagnosis. This report also highlights a chromosome region potentially involved in autistic disorders.

## Background

According to the orientation of the duplicated segment, duplications may be classified either as tandem or inverted, being the last usually associated with deletion of the distal region of the duplicated chromosome [1]. The best studied cases of inverted duplications (inv dup) are the inv dup(8p) [2,3] and bisatellited inv dup(15) [4], which are usually non-mosaic. In contrast, mosaic inverted duplications are derived from different post-zygotic mechanisms for which various possible origins have been proposed [5-7]. There is also a particular subset of inv dup in which the duplication ends terminally on the chromosome and which are named terminal inv dup [8,9].

Tetrasomy of distal 3q segments is associated with adverse phenotypic manifestations, ranging from mild developmental delay to deep facial dysmorphisms, resembling patients with the dup(3q) and Brachmann-de Lange syndromes. Accordingly, some of the patients with 3q tetrasomy show hirsutism, synophrys, broad nasal root, anteverted nares, thin upper lip with downturned mouth corners, craniosynostosis, urinary tract anomalies, micrognathia, cleft palate and malformed ears, characteristics also seen in patients with the dup(3q) syndrome [10]. Brachmann-de Lange syndrome (BDLS) has overlapping features with dup(3q) syndrome, but with apparently normal chromosomes [11,12].

In this study we characterize by molecular cytogenetics a case of an autistic child previously reported by our group, with a mosaic partial tetrasomy of a distal chromosome 3q segment translocated to the short arm of the chromosome 12 [13]. To our knowledge, this is the first report of a mosaic terminal inv dup(3q) captured in an intact 12p subtelomere. Using fluorescence *in situ *hybridization (FISH) and array-based comparative genomic hybridization (aCGH) we have better characterized the extra material of chromosome 3 as qter → q26.31::q26.31 → qter. Furthermore, the mechanism of formation for this rearrangement is discussed.

## Case presentation

The male child was the first born of non-consanguineous healthy parents and was delivered at term after an uneventful pregnancy. His birth weight was 2850 g (<5th centile) and there were no major neonatal problems, except for the club foot surgically corrected. However, a general developmental delay was noted soon after birth and fever convulsions were observed between the age of 9 and 24 months old.

As a consequence of suspicions of a pervasive developmental disorder of autistic type, at the age of 3, the proband was referred to a child psychiatrist, who directed him to an autism unit. At 11 years old, his height was between the 25th and 50th centile, weight was on the 90th centile and head circumference was on the 50th centile. Some minor dismorphic features were also observed, such as low set and slightly enlarged ears, high and arched palate and round face. No skin pigmentation disorders were observed. He was submitted to a multidisciplinary neurodevelopmental assessment and showed an adaptive behavior at the 30 month age level and a nonverbal IQ of 61, corresponding to a mild delay, a diagnosis of autism was based on Autism diagnostic interview-revised and the Statistical Mental Disorder IV edition criteria [13-15]. At adult age the proband maintain a clinic of autism and has an adaptative behaviour evaluated with Vineland Adaptative behaviour scales – survey form with a standard score of 30 for communication domain, 59 for daily living skills domain and 63 for socialization domain [16].

## Results

### Conventional cytogenetic analysis

**Figure 1:**
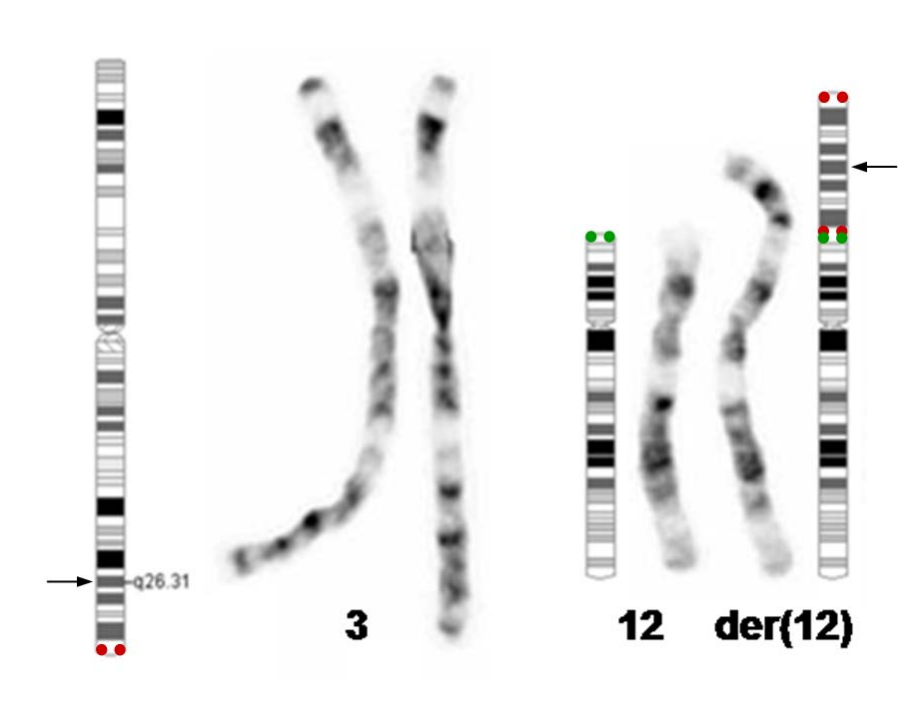
**Partial GTG-banded karyogram and ideograms**. GTG-banded normal chromosomes 3 homologous (left) and normal chromosome 12 plus the der(12) (right) are showned. Ideograms of the normal chromosome 3, 12 and of the der(12) with the inv dup(3)(q26.31qter) segment translocated on 12pter are represented. The localization of subtelomeric probes 3qter (red) and 12pter (green) are signed in the ideograms. The breakpoint of 3q26.31 is represented by an arrow (→).

The GTG banded chromosome analysis on peripheral blood lymphocytes of the proband showed a normal cell line 46,XY in 44% of the metaphases studied. In the other 56% of cells, an unbalanced karyotype with additional material on the terminal end of the short arm of chromosome 12 was identified as 46,XY,add(12)(p13.3) (Fig. 1). The banding pattern suggested that if any portion of chromosome 12 could be lost, it would be a very small region of the tip of 12p. The additional material on chromosome 12 could not be easily matched to a specific region of either chromosome 12 or any other chromosome.

Skin fibroblast cultures of the proband confirmed the mosaic, with the abnormal cell line in only 14% of the 50 metaphases studied. As expected, the karyotypes of both parents were normal.

### Molecular cytogenetic Studies

#### FISH and MCB analysis

**Figure 2:**
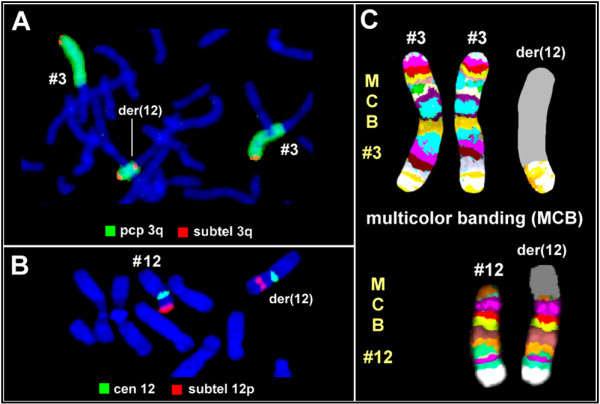
**FISH analysis and multicolour banding pattern**. **A) **Partial chromosome 3q paint (pcp 3q-green) of normal chromosomes 3 and of der(12), merged with 3q subtelomeric probe that hybridized at both ends of the additional segment (red). **B) **Hybridization of the subtelomeric probe 12p on the normal and the derivative chromosome 12. **C) **Multicolor banding analysis using specific probe sets for chromosomes 3 and 12. The resultant pattern proved that the additional material on der(12) resulted from an inverted duplication of the terminal portion of 3q.

In order to identify the extra material on the der(12), FISH was performed using the whole chromosome paint (wcp) M-multiprobe system (Cytocell), with specific libraries for all chromosomes. The wcp12 did not paint the entire 12p, showing that the extra material was not derived from chromosome 12 (data not shown). Also, the wcp12 did not paint any portion of any other chromosome ruling out a reciprocal translocation. When wcp3 was used, both chromosomes 3 were fully painted and an additional signal was present on one of the 12p-arms (data not shown). On the re-evaluation of the case partial chromosome paint (pcp) 3q was used showing that the additional material was derived from the long arm of chromosome 3 (Fig. 2A). The combined results of G-banding and FISH indicated that only the distal part of 3q could be involved in the rearrangement, suggesting a partial trisomy of 3q25? → qter.

Further FISH analysis was performed using specific probes for the subtelomeric regions of chromosome 3 (D3S4559, D3S4560; Vysis) which showed that the subtelomeric regions of both chromosomes 3 were intact, and that the 3q subtelomeric probe hybridised at both ends of the extra segment, suggesting an inverted duplication of the terminal 3q (Fig. 2A). To identify a possible loss or cryptic translocation of the 12p sutelomere, the subtelomeric probe for 12pter (GenBank U57865, Vysis) was used. It hybridized as expected in the normal chromosome 12 and an hybridization signal was also seen in the der(12), at the junction point of the additional material (Fig. 2B). The G-banding was revisited, taking into account the FISH findings (Fig. 1), and the additional material on 12p was interpreted as an inverted duplicated segment inv dup(3)(qter → q26.3::q26.3 → qter) being the patient tetrasomic for the region 3q26.3 → 3qter.

To confirm this result described previously by us [13], multicolor banding (MCB) analysis was performed using a chromosome 3 specific probe set [17]. It was proved that the additional material on 12 resulted from an inverted duplication of the terminal portion of 3q(:qter → q26.3::q26.3 → qter) (Fig. 2C). Altogether these results led to the final karyotype: mos 46,XY,add12(p13.3).ish inv dup(3)(qter → q26.3::q26.3 → qter)(wcp3+,pcp3q+,D3S4560++) [56]/46,XY [44].

#### aCGH Analysis

**Figure 3:**
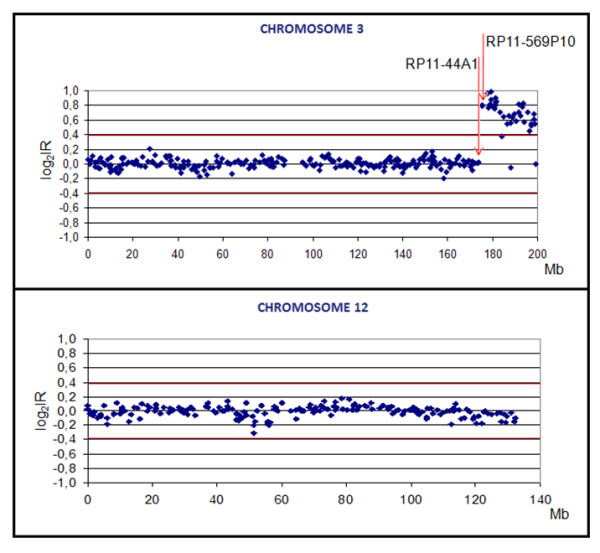
**Result of the array CGH analysis for chromosomes 3 and 12**. The Y-axis represents the log_2 _of the intensity ratios (log_2_IR) of the combined dye swap experiments of patient/control DNA. In the X-axis the distance of the BAC clones from the p telomeres is indicated (Mb). The red lines are the thresholds (4 × S.D.) for clone deletion (-0.39) or clone amplification (+0.39). The first clone with amplification is RP11-569P10 (starting at 174.37 Mb) and the flanking clone is RP11-44A1 (ending at 173.86 Mb).

The BAC-based aCGH performed in DNA extracted from peripheral blood of the proband revealed an amplification from 174.37 Mb (3q26.31) to 199.29 Mb (3qter), and absence of a deletion on chromosome 12 (Fig. 3). These observations were important to characterize the extension of the extra material derived from chromosome 3 and to exclude a possible deletion in 12pter.

## Discussion

In this work we have characterized by molecular cytogenetics a tetrasomy of a 3qter fragment. The carrier presents minor facial dysmorphisms and general developmental delay associated with an autistic disorder. His karyotype was initially established from peripheral blood lymphocytes as mos 46,XY,add(12)(p13.3) [56]/46,XY [44] [13]. The use of MCB and aCGH techniques allowed the characterization of the extra material as being derived from chromosome 3, involving an inv dup(3)(qter → q26.3::q26.3 → qter) and with a length of 24.92 Mb.

**Figure 4:**
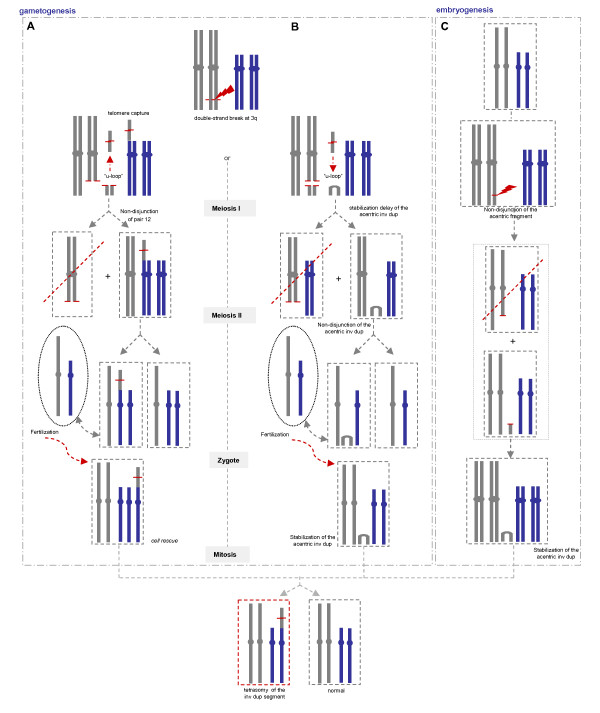
**Schematic representation showing the three mechanisms proposed for the formation and stabilization of the terminal inv dup 3q**. **A-B) **Two possible mechanisms having in common a first step in gametogenesis with a double-strand break event at 3q, repaired by fusion of the two sister chromatids (U-loop) and giving rise to an acentric inv dup; **A) **Subsequent step would be the telomere capture from chromosome 12, a non-disjunction of the 12 homologous at meiosis I, leading to a gamete that after fertilization originated a trisomic zygote, that by *cell rescue *generated two cell lines; **B) **Other alternative mechanism would be the post-zygotic stabilization delay of the acentric inv dup; **C) **The third possibility is the generation of a terminal acentric inv dup as a result of a post-zygotic event at embryogenesis, which would necessarily lead to mosaicism.

Inverted duplications are a kind of genetic lesions that can appear either as mosaic, or non-mosaic, depending on the time that they are formed [18]. There are different well established mechanisms proposed for the origin of terminal inv dup. The present case, however, does not adjust entirely with the usual mechanisms proposed for terminal inv dup that would imply a concomitant deletion or a distal extra marker chromosome stabilized by neocentromerization. We propose three alternative mechanisms (Fig. 4A–C), two of them could have in common a first step in gametogenesis with a double-strand break event at 3q, repaired by fusion of the two sister chromatids (U-type exchange) and giving rise to an acentric inv dup (Fig. 4A–B). One possible mechanism for the subsequent step would be the healing by telomere capture from a non-homologous chromosome 12. A non-disjunction of the 12 homologous at meiosis I, led to the formation of a gamete that after fertilization originated a trisomic zygote, which included the der(12). In the first mitotic division, in an attempt of cell rescue, two cell lines would have been generated, by loosing either the der(12) or the normal 12 chromosome. A consequence of the heterodisomy generated by the segregation error at meiosis I could be a chromosome 12 uniparental disomy [19]. The other alternative mechanism (Fig. 4C) would be the generation of a terminal acentric inv dup either as a result of a post-zygotic mitotic division, which would necessarily lead to mosaicism, or at meiosis. In this case, the post-zygotic stabilization delay of the acentric inv dup, associated with the telomere capture from a non-homologous chromosome 12, could explain the mosaicism [7,20,21].

Vanneste and colleagues have described that post-zygotic chromosome instability is highly frequent in cleavage-stage embryos, leading to segmental chromosomal imbalances and mosaicism, probably a common cause of constitutional chromosomal disorders. In this study, fifty-five percent of embryos carried terminal segmental imbalances, that were the result of DNA double-stranded breaks possibly followed by non-disjunction of the acentric fragment [22]. This study reinforces the theory that mosaic inv dup formation is a post-zygotic event.

There are only twelve reported cases in the literature of inv dup associated with tetrasomy for distal chromosome 3q [10,23-33]. Of all documented cases, our proband is the only one in which the tetrasomy is not an intrachromosomal triplication [32,33] or a supernumerary marker chromosome [10,23-31].

The chromosome region of the present rearrangement has been reported to be involved in both the BDLS (q26.3-q27) and the dup(3q) syndromes (with q26.3 being the critical region) [34-37]. Nonetheless, besides the mental retardation, the low set ears, the arched palate, our patient does not have any of the other 23 physical features compiled for those syndromes by Faas and colleagues [34].

Taking into account the twelve cases previously reported and the present one, it becomes evident that the phenotypes associated with tetrasomy of distal 3q segments are heterogeneous [10,23-33]. As a consequence, we failed to establish any genotype-phenotype correlation once neither the region involved nor the degree of the mosaicism could be correlated with a consistent pattern. Nevertheless, the presence of skin pigmentary disorders is a particular feature that connects the majority of the cases reported [23,25,27-32]. Indeed, hyperpigmentation is present in 8 of the reported patients, with a pattern concordant with lines of Blaschko in 5 patients. Correlating these cases, Gimelli and colleagues proposed the presence of a gene involved in skin pigmentation defects located at 3q27.1-qter region [30]. However, hypopigmentation and hyperpigmentation following the Blaschko's lines are relatively common in individuals with chromosomal mosaicism. In the present case, although involving the 3q27.1-qter region, there are no skin pigmentary alterations.

The autistic behavior observed in our patient, and not reported in any other case with distal 3q tetrasomy, is an interesting feature. According to the results of a genome-wide screen performed by Auranen and colleagues, there is evidence for a major susceptibility locus on chromosome 3q25-q27 for the autism-spectrum disorders [38]. Accordingly, a study of the same group revealed the existence of an allelic association on chromosome 3q25-q27 in families with autism spectrum disorders originating from a sub-isolate of Finland [38,39]. Since no other reported patient with 3qter tetrasomy demonstrated autistic behavior, we could be facing a random occurrence. However, it would be interesting to evaluate children with autistic behavior for micro-rearrangements in this region of chromosome 3.

To the best of our knowledge, this is the first study describing a mosaic interchromosomal inverted duplication of a 3qter segment captured in a non-homologous intact subtelomere (12pter). Also new is the fact that the proband presents an autistic behavior, not observed in any other patients with the same genomic imbalance. The concomitant employment of aCGH and multicolor FISH techniques contributed to the understanding of this unusual rearrangement.

## Methods

### Cytogenetic and FISH studies

Cytogenetic analysis was carried out on GTG-banded chromosomes (650 bands per haploid genome) prepared from peripheral blood lymphocytes and fibroblast cultures according to the standard protocols [40].

FISH studies were performed on metaphase spreads according to the standard procedures. M-multiprobe system (Cytocell Ltd, Adderbury, UK) was used to paint all chromosomes. For chromosome 3 individual wcp with a chromosome 3-specific library (Vysis Abbott Molecular, Inc., Des Plaines, IL) was used as well as specific subtelomeric probes for chromosomes 3 and 12 (Vysis). The derivative chromosome 12 was also studied by MCB applying the probe sets for chromosome 3 and 12, as described by Liehr and colleagues [17]. FISH results were analyzed using a Nikon Eclipse fluorescence microscope (Nikon Instruments Europe B.V., Badhoevedorp, The Netherlands) coupled with a Cytovision system (Applied Imaging International Lda, Newcastle upon Tyne, UK). MCB was analysed using a Zeiss Axioplan fluorescence microscope (Zeiss; Jena, Germany) with MetaSystems (Isis) software (Altlussheim, Germany).

### BAC aCGH

BAC aCGH was performed for all genome screening using a 1 Mb clone set. Control and patients' DNA were labelled with Cy5 and Cy3 dCTP's (Amersham Pharmacia Biotech, Piscataway, New Jersey) using a random prime labelling system (Bioprime DNA Labelling System, Invitrogen, Carlsbad, CA) according to established protocols [41]. Scanning of the array was performed at 532 nm and 635 nm using a GenePix4000B scanner (Axon Instruments) and images were analyzed with the GenePix Pro 6.0 software. Correction of spot intensities for the local background followed previously described protocols [42].

## Competing interests

The authors declare that they have no competing interests.

## Authors' contributions

IMC, drafted the manuscript and coordinated the study.

JBM, performed the array-CGH analysis and has been involved in the drafting of the manuscript.

CR, provided valuable support.

AW and NK, carried out the application of multicolor banding.

LB and JRV, were involved in array-CGH analysis.

GO, provided the clinical data and biological samples.

EM, performed the cytogenetic studies as well as FISH experiments and has been involved in the drafting of the manuscript.

All authors read and approved the final manuscript.
